# Snail Overexpression Alters the microRNA Content of Extracellular Vesicles Released from HT29 Colorectal Cancer Cells and Activates Pro-Inflammatory State In Vivo

**DOI:** 10.3390/cancers13020172

**Published:** 2021-01-06

**Authors:** Izabela Papiewska-Pająk, Patrycja Przygodzka, Damian Krzyżanowski, Kamila Soboska, Izabela Szulc-Kiełbik, Olga Stasikowska-Kanicka, Joanna Boncela, Małgorzata Wągrowska-Danilewicz, M. Anna Kowalska

**Affiliations:** 1 Institute of Medical Biology, Polish Academy of Sciences, 93-232 Lodz, Poland; pprzygodzka@cbm.pan.pl (P.P.); dkrzyzanowski@cbm.pan.pl (D.K.); ksoboska@cbm.pan.pl (K.S.); iszulc@cbm.pan.pl (I.S.-K.); jboncela@cbm.pan.pl (J.B.); 2 Faculty of Biology and Environmental Protection, University of Lodz, 90-236 Lodz, Poland; 3 Department of Diagnostic Techniques in Pathomorphology, Medical University of Lodz, 90-419 Lodz, Poland; olga.stasikowska@umed.lodz.pl (O.S.-K.); malgorzata.wagrowska-danilewicz@umed.lodz.pl (M.W.-D.); 4 Department of Pediatrics, The Children’s Hospital of Philadelphia, Philadelphia, PA 19104, USA

**Keywords:** Snail transcription factor, extracellular vesicles, colon cancer, pre-metastatic niche

## Abstract

**Simple Summary:**

Knowledge of the factors that help migration of carcinoma cells is important for prevention of metastasis. Cancer cells release small particles, extracellular vesicles (EVs) that contain such factors. The aim of this study was to assess if the content of EVs changes through different stages of colorectal cancer (CRC) and evaluate how this process affects cancer progression in vivo in mouse CRC model. We found that EVs released from cells that have migratory properties contain different factors then EVs released from original tumor cells. We also show here that EVs can be incorporated into other cells that facilitate metastasis and change their properties depending on the EVs content. The content of cell-released EVs may also serve as a biomarker that denotes the stage of CRC and may be a target to prevent cancer progression.

**Abstract:**

During metastasis, cancer cells undergo phenotype changes in the epithelial-mesenchymal transition (EMT) process. Extracellular vesicles (EVs) released by cancer cells are the mediators of intercellular communication and play a role in metastatic process. Knowledge of factors that influence the modifications of the pre-metastatic niche for the migrating carcinoma cells is important for prevention of metastasis. We focus here on how cancer progression is affected by EVs released from either epithelial-like HT29-cells or from cells that are in early EMT stage triggered by Snail transcription factor (HT29-Snail). We found that EVs released from HT29-Snail, as compared to HT29-pcDNA cells, have a different microRNA profile. We observed the presence of interstitial pneumonias in the lungs of mice injected with HT29-Snail cells and the percent of mice with lung inflammation was higher after injection of HT29-Snail-EVs. Incorporation of EVs released from HT29-pcDNA, but not released from HT29-Snail, leads to the increased secretion of IL-8 from macrophages. We conclude that Snail modifications of CRC cells towards more invasive phenotype also alter the microRNA cargo of released EVs. The content of cell-released EVs may serve as a biomarker that denotes the stage of CRC and EVs-specific microRNAs may be a target to prevent cancer progression.

## 1. Introduction

Colorectal cancer (CRC) is the third most common cancer in men and second in women worldwide. It is predicted that the number of deaths will rise to 1.1 million per year worldwide by 2030 [1]. Formation of distant metastases are the primary cause of CRC treatment failure and patient death [2]. In the metastasis process, to enter the circulation, cancer cells lose their epithelial features, exhibit decreased polarity and intercellular adhesion, undergo cytoskeletal reorganization and become more motile. This phenotypic transformation of epithelial carcinoma into mesenchymal-like cells (EMT) is triggered by several factors including Snail transcription factor that plays crucial role at the beginning of the EMT process [3].

Crucial for cancer progression is also the intercellular cross-talk and subsequent regulation of both local and distant microenvironments. Kaplan et al. [4] introduced the concept termed “pre-metastatic niche” that is defined as the microenvironment that facilitates the formation of metastasis and consists of immune and stromal cells and also the components of the primary tumour. A number of niche-promoting molecules has been identified in various metastasis mouse models [5]. Monocytes and macrophages associated with tumour environment have been widely recognized as immunological effectors [5]. Macrophages display functional plasticity in response to local microenvironment stimuli and participate in cancer-related inflammation, matrix remodelling, immune escape and ultimately in cancer metastasis. Additionally, cancer cell-derived extracellular vesicles (EVs) are recognized as significant contributors to different aspects of modifications of pre-metastatic niches [6]. 

EVs are defined as a heterogeneous group of a phospholipid bilayer particles that are released to the extracellular environment by most cells in the organism and are present in all body fluids [7]. EVs can be divided into main three subpopulations including exosomes (exo), microvesicles/ectosomes (MVs) and apoptotic bodies (APOs) that differ in size, formation process and content [8]. EVs contain a variety of bioactive molecules, including proteins, lipids and multiple nucleic acid species, including the most extensively studied class of microRNA (miRNA/miR) [6,9]. The cargo of EVs may either reflect the cell of origin or can be actively sorted [10,11]. Recent reviews summarize the importance of EVs as communicators between cells that accelerate cancer progression and metastasis [12,13]. EVs can stimulate angiogenesis, matrix remodelling and modulation of immune response [9,14,15,16]. Factors, including cytokines, chemokines and EVs that are released from cancer or other cells that are linked to inflammation, influence the environment of pre-metastatic niches in distant organs [17].

We have previously shown that Snail, as an early regulator of EMT, affects the transcriptome and miRNA profile of human HT29 CRC cells and changes HT29 cells phenotype to pro-migratory [18,19]. Here we show that HT29 cells overexpressing Snail, in comparison to epithelial-like HT29 cells, release EVs with different miRNA content and postulate that HT29-Snail-EVs modify the activity of cells constituting the pre-metastatic niche, thus facilitating cancer progression.

## 2. Results

### 2.1. Characterization of Extracellular Vesicles (EVs) Released from Control HT29 and HT29-Snail Cells

#### 2.1.1. Size and Purity

**Figure 1:**
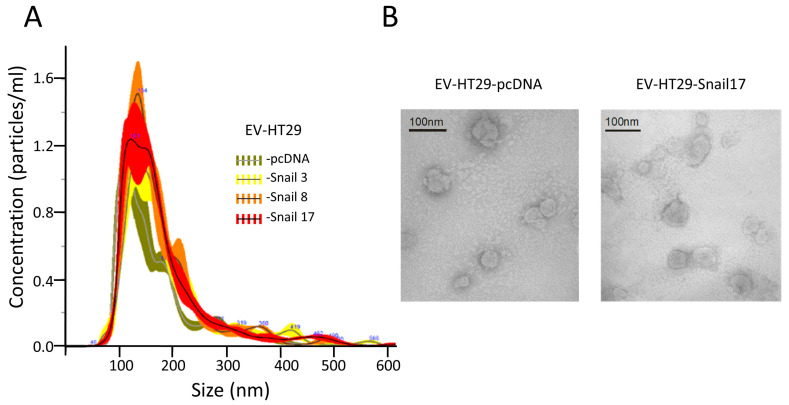
Characterization of extracellular vesicles (EVs) released by HT29 clones. EVs were isolated from conditioned media after 24 h of incubation in FBS-free culture media of HT29 clones. (**A**) NTA showed similar numbers and sizes of EVs isolated from HT29 clones. (**B**) Representative Electron microscopic image of EVs derived from HT29-cDNA and HT29-Snail 17. Scale bar, 100 nm.

The preparation of EVs free of cellular debris, FBS-derived vesicles or any non-EVs RNAs and proteins is critical for any analyses. We applied a commonly used procedure for isolation of EVs that we used in a previous study [18,19]. Cells release vesicles that according to current nomenclature can be classified as exosomes (exo, 30–120 nm), microvesicles (MVs, 0.1–1.0 μm) and apoptotic bodies (0.8–5.0 μm) [20]. We purified EVs that are the mixture of exo and MVs. EVs sizes were similar for all HT29 clones with sizes of 80 to 240 nm (Figure 1A).

Similar vesicles number (~5.2 × 10^13^) for each preparation of EVs were isolated from the same number of cells (~6.8 × 10^8^). The spheroid morphology of EVs as well as their size were confirmed by TEM (Figure 1B). EVs were enriched by markers such as CD63, CD9 and flotillin-1 as compared to lysates of cells of origin (Figure S1). They also contained 70 kDa heat-shock protein (HSP70) and were Annexin V positive; the purity of EVs was confirmed by the absence of cytochrome c and GM130 (Figure S1).

#### 2.1.2. miRNA Content

**Figure 2:**
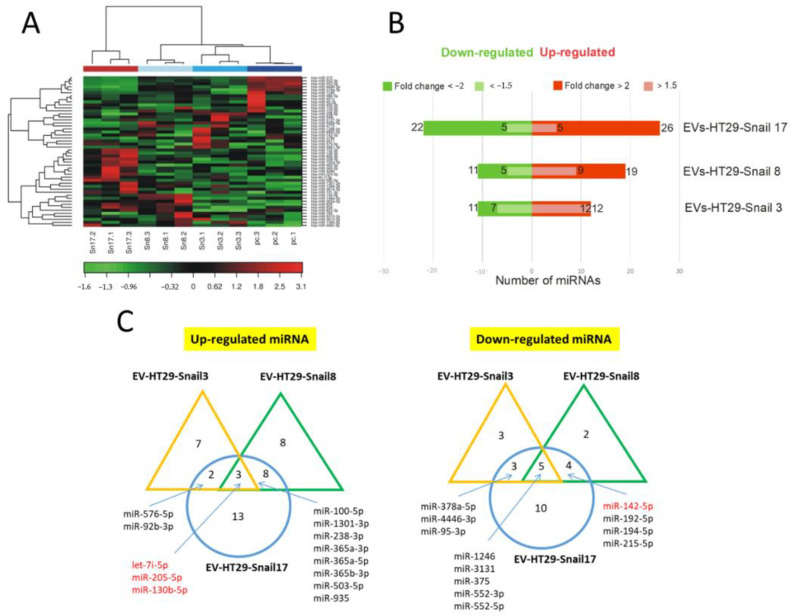
Changes in the expression of miRNAs in EVs released by HT29-Snail cells as compared to EVs from HT29-pcDNA cells. (**A**) Heatmap and unsupervised hierarchical clustering by sample and miRNA. (**B**) Number of differentially expressed miRNAs detected by NGS analysis, that were either significantly upregulated (red) or downregulated (green) in EVs derived from HT29-Snail-3, -8 and -17 versus EVs from control cells. (**C**) Venn diagrams show differentially expressed miRNAs. The most regulated miRNAs in all three or two clone-EVs are marked. (corrected FDR *p* < 0.05 and fold change > 2). miRs marked in red are discussed in the text.

The miRNA profiling was performed by Next Generation Sequencing (NGS) analysis of the total mRNA isolated from EVs. There were no significant differences in the number of detectable miRNAs between EVs of control and either of HT29-Snail clones (Figure S2). Further, the two-way hierarchical clustering of miRNA was performed (Figure 2A). As presented in Figure 2B,C, we identified 23 miRNAs differentially expressed in EVs released from HT29-Snail 3, 30 miRNAs from HT29-Snail 8, and 48 miRNAs from HT29-Snail 17.

Three miRNAs: let-7i, miR-205, and miR-130b were up-regulated while five miRNAs: miR-1246, miR-3131, miR-375, miR-552-3p and miR-552-5p were down-regulated in HT29-Snail-derived EVs (Figure 2C). Additionally, as it is shown in Table S1, among other miRNAs altered more than two times in EVs released from HT29-Snail clones, miR-483-5p (HT29-Snail 17-EVs) was upregulated and miR-142-5p (HT29-Snail 8, -17-EVs) as well as miR-203a and miR-203b-3p (HT29-Snail 17-EVs) were downregulated. Moreover, as shown in Table S2, there were also miRNAs upregulated in EVs released from HT29-Snail cells with a lower fold change. Those differentially expressed miRNAs (marked in red in Tables S1 and S2) were reported to be important in cancer progression as pointed out in the Discussion section.

#### 2.1.3. Gene Ontology (GO) Enrichment Analysis

To identify the biological processes that might be triggered in the response to elevated Snail levels in the HT29 clones that are shedding EVs, Gene Ontology (GO) enrichment analysis was performed to identify GO terms that are significantly associated with differentially expressed microRNAs in EVs released by the clones. Positive regulation of thyrosine phosphorylation of STAT-3 and -1 was at the top of Biological Process in the GO analysis (Figure S3).

### 2.2. In Vivo Studies

**Figure 3:**
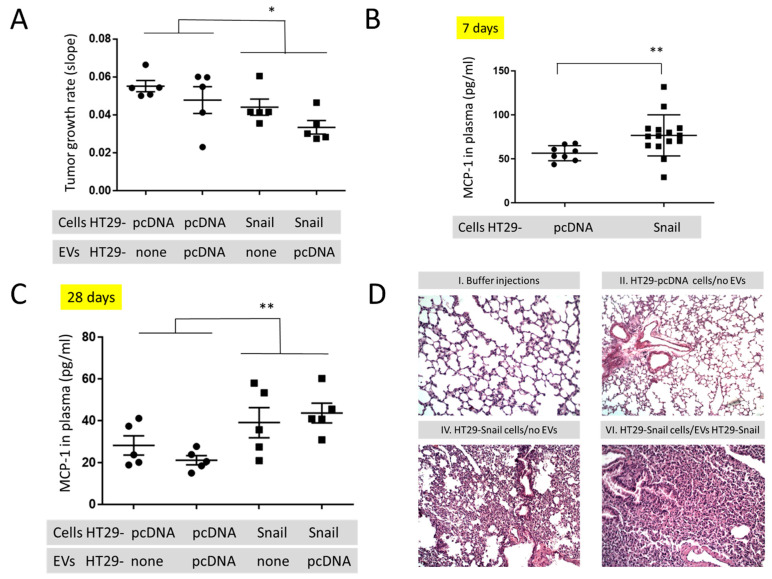
In vivo mouse studies in animals injected s.c. with HT29-pcDNA- (small dot) or HT29-Snail17- (small cube) cells. (**A**) The growth rate of tumours (day 12–28) in animals with HT29-pcDNA or HT29-Snail administered s.c. and additionally injected i.v. with EVs released by HT29-pcDNA, mean value ± SEM: 0.052 ± 0.04 and 0.039 ± 0.03, respectively (**B**) Levels of monocyte chemoattractant protein-1 (MCP-1/CCL2) measured in the plasma of mice at day 7 (before first injection of EVs). (**C**) Levels of monocyte chemoattractant protein-1 (MCP-1/CCL2) measured in the plasma of mice at day 28 (end of experiment). Mean value ± SEM for animals injected s.c. with HT29-pcDNA and HT29-Snail were 24.8 ± 2.7 and 41.5 ± 4.1, respectively. (**D**) Representative H&E staining of murine lungs. 100× magnification. The images of groups IV and VI (see Table 1) show interstitial pneumonia pictures. *n* = 10–15, * *p* < 0.02; ** *p* < 0.01.

To look for the in vivo effect of Snail on the cancer progression we injected athymic mice subcutaneously (s.c.) with the same number (1.5 × 10^6^/mouse) of control (HT29-pcDNA) and HT29-Snail 17 cells. Clone 17 contains the most elevated Snail expression ([18] and Figure S5), and their EVs have the highest number of differentially expressed miRNA (Figure 2 and Tables S1 and S2). Further, animals of both groups were injected intravenously (i.v. day 8, 12, 15 and 18) with the same amount (10 μg/mouse) of HT29-pcDNA-EVs, and observed for 28 days. Administration of HT29-pcDNA-EVs did not have statistical impact on neither tumour growth nor plasma MCP-1/CCL2 levels (Figure 3A,C, Table S3) within the groups of mice injected with either HT29-pcDNA cells or HT29-Snail cells. However, there was a ~20% difference in the growth rate of tumour (day 12 to 28) between the control and HT29-Snail mice, regardless of the injection of EVs (Figure 3A). Growth of tumours in mice administered s.c. with HT29-Snail cells was slower in comparison to administration of control cells, which is not surprising since HT29-Snail cells are already in the early EMT stage [18,19]. We have also observed an increase in the concentration of monocyte chemotactic protein-1 (MCP-1/CCL2) at day -7 (Figure 3B) and -28 (Figure 3C) in the plasma of mice that bore a tumour induced by injection of HT29-Snail17 cells as compared to control mice injected with HT29-pcDNA cells. We did not observe any metastatic sites of HT29 cells in the lungs, livers or intestines after 28 days of tumour growth. However, early interstitial pneumonias were observed after 28 days in animals injected with HT29-Snail17 cells as compared to animals injected with HT29-pcDNA (Table 1, group IV and II, respectively). Injection of EVs released from control HT29-pcDNA cells slightly increased the number of mice with lung infection in control group of mice that bore a tumor induced by injection of HT29-pcDNA cells (Table 1, group II and III). Based on the fact that HT29-Snail cells injected s.c. into mice may secrete additional HT29-Snail-EVs, we have additionally injected such EVs into the group of mice bearing tumors induced by HT-29-Snail cells. We did not observe any changes in either tumor growth or MCP-1/CCL2 levels (Table S3). However, in this group (Table 1, group VI) we observed the highest number of animals presenting lung inflammation (80%) As presented on Figure 3D, inflammatory infiltration from lymphocytes and thickened interalveolar septum were observed in lungs of those mice.

### 2.3. In Vitro Studies

#### 2.3.1. Uptake of EVs by THP-1 Derived Macrophages (TDM)

**Figure 4:**
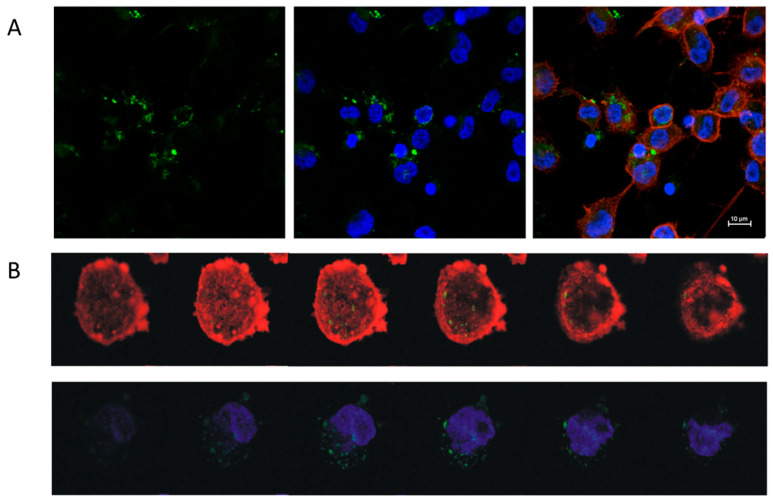
Uptake of EVs derived from Snail-HT29 cells (clone 17) by TDM. HT29-Snail EVs were labeled with PKH67 dye (green). Nuclei were stained with Hoechst (blue), actin was stained with Phalloidin Texas Red (red). (**A**) Representative confocal laser scanning microscope. (**B**) Z-stack analysis of cells and incorporated EVs. Note: Viability of TDM cells was not affected by the incorporation of EVs (Figure S4).

The uptake of EVs by TDM was visualized by confocal imaging (Figure 4A,B). EVs obtained from either control HT29-pcDNA or HT29-Snail clones -3, -8 and -17 were incorporated into TDM in FBS-free media for 4 h. The viability of TDM cells was not changed by incorporation of any EVs (Figure S4).

#### 2.3.2. Effect of EVs Released from HT29 Clones on Macrophage Activity

**Figure 5:**
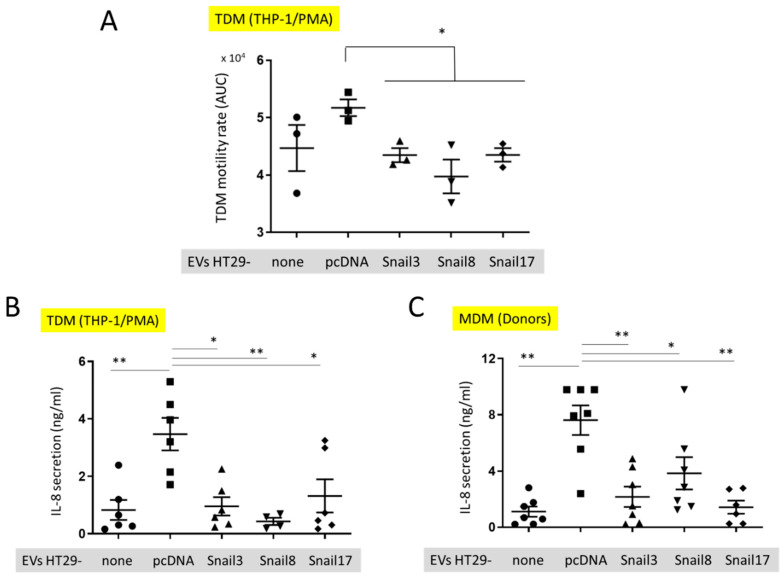
In vitro effect of EVs on macrophages. (**A**) Motility of TDM cells before (small dot) after the uptake of EVs isolated from control HT29-pcDNA (small cube) or HT29-Snail cells (clone 3- small triangle, clone 8- small upside triangle and clone 17- diamond). (**B**) IL-8 release from TDM after treatment with EVs indicated. (**C**) IL-8 release from macrophages isolated from healthy donors blood (MDM) and incubated with EVs. *n* = 3–7, * *p* < 0.05; ** *p* < 0.01.

We examined whether the incorporation of EVs released by HT29-Snail cells that represent the intermediate state of EMT, affects the activity of macrophages differently than incorporation of EVs released from epithelial-like HT29 cells (Figure 5).

The motility of TDM was evaluated by measuring the cell speed changes in time. There was a slight, but not significant, increase in the motility of TDM that had incorporated EVs released from HT29-pcDNA as compared to control TDM cells (Figure 5A). However, the motility of TDM cells with incorporated EVs released from HT29-Snail cells was inhibited in comparison to cells containing HT29-pcDNA-EVs. (Figure 5A). We have also quantified the level of cytokines secreted by TDM. We observed a significantly increased level of IL-8 released by TDM (Figure 5B) treated by EVs released from HT29-pcDNA cells, as compared to non-treated TDMs (no EVs). However, incorporation of HT29-Snail-EVs resulted in decreased IL-8 levels (Figure 5B). Next, we performed the analysis of cytokines released by macrophages derived from monocytes isolated from healthy donors and differentiated with GM-CSF (MDM). Consistent with the previous results, MDM cells incubated with EVs released from HT29-Snail clones secreted less IL-8 as compared to cells incubated with control HT29-pcDNA-EVs (Figure 5C).

## 3. Discussion

Our group has recently reported that overexpression of Snail drives HT29 colon cancer cell to a partial-EMT and modulates the expression of specific protein transcripts and miRNAs [18,19,21]. We reasoned that cancer cells at various EMT stages will release extracellular vesicles of different content and thus differently affect recipient cells. Changes in the protein levels in EVs released from cells stimulated by Snail were already observed [22]. As a rule, the cargo loaded into EVs reflects the status of cancer cells [23], but potential preferential packaging into EVs has also been suggested [11]. The most extensively studied class of factors transported between cells by EVs are miRNAs that regulate the translation of target mRNAs in recipient cells [10,24]. We show here that overexpression of Snail in HT29 cells significantly triggers changes on individual miRNAs levels.

We are aware of the fact that our HT29-Snail clones differ in the amount of Snail overexpressed in their cells. In this study, we used several HT29-Snail clones with clone 3 representing the lower and clone 17 the highest Snail level [18]. Thus, each of the clones may reflect a slightly different EMT stage.

In EVs released from all three HT29-Snail clones let-7i, miR-205 and miR-130b-5p miRNAs were highly upregulated. There are conflicting reports concerning the role of miR-205 and let-7i in cancer [25,26,27]. Whether particular miRNA is considered as tumor suppressor or onco-miRNA appears to be dependent on the specific cancer and tumor-environment [28,29]. Increased migratory properties of HT29-Snail cells with elevated miR-205 and let-7i expression was shown previously by us, pointing on their role in CRC progression [19]. The mRNA targets for miR-205-5p and let7i-5p were also shown in our previous study. Potentiated miR-205 expression was correlated with Dynamin 3 (DNM3) mRNA decrease. DNM3 is considered as a cancer suppressor. Thus, from the tumour point of view redundant during progression of the disease and decreased in HT29-Snail cells [19].

We observed miR-130b-5p enrichment in EVs released from all our HT29-Snail clones. In contrast to miR-205 and let-7i up-regulation in EVs, which mirrored intracellular changes in miRNAs expression, miR-130b-5p was not up-regulated in HT29-Snail cells [19]. This suggests that miR-130b-5p could be actively sorted into vesicles released from cells that overexpress Snail and thus undergo EMT. MiR-130b was identified in the microvesicles of leukemia K562 cells, but, unlike in CRC cells in this study, at an equal expression level as in cells of origin [30]. In various human tumor types altered miR-130b expression has been implicated as either promoting or suppressing tumorigenesis; miR-130b is significantly downregulated in pituitary adenomas and endometrial cancer [31,32] whereas it is upregulated in bladder cancer, melanoma, metastatic renal carcinoma [33,34,35]. The contribution of miR-130b to CRC progression is also the subject of the debate. In cell lines SW-480 and SW-620 over-expression of miR-130b downregulates integrin β1, leading to the impaired migration and invasion of CRC cells [36] whereas another analysis of a series of CRC cell lines showed that miR-130b acts as an efficient inducer of EMT in vivo and in vitro, likely through up-regulation of Snail and ZEB1 transcription factors. The effects of miR-130b on promoting cell migration and invasion of CRC cells with poor prognosis for colorectal cancer was also indicated [37]. Whether the miR-130b that is packed into EVs released from HT29-Snail clones affects positively colorectal cancer progression is not definitely comprehensible from our studies. However, our in vivo studies (Table 1) suggest that the presence of EVs released from CRC cells that undergo EMT and contain packed miR-130b may lead to increased lung inflammation that facilitates cancer progression. This is in agreement with the previously identified effects [37] and suggests that miR-130b may be a target to attempt to slow the CRC progression. Additionally, the presence of miR-130b on EVs released from CRC cells can serve as a biomarker of an advanced stage of CRC.

We found increased amount of miR-483-5p in EVs released from HT29-Snail clone 17 that express the highest amount of Snail and was used in our in vivo studies. Increased content of miR-483-5p was observed in exosomes isolated from plasma of a CRC-patient in various stages and was found in EVs from SW480 CRC line suggesting its diagnostic potential [38]. We have also observed that miR-221, miR-222, and miR-125a were overexpressed in HT29-Snail-EVs, although with lower fold change. MiR-221 is among commonly upregulated miRNAs in CRC in tumors [39] and in patient’s serum [40] while miR-222-3p promotes macrophage polarization and differentiation to M2 phenotype in vitro and in vivo, which enhance the progression of epithelial ovarian cancer [41]. Additionally, the decrease in miRNA-34a and miRNA-203b in EVs released from HT29-Snail confirms the previously described reports about repression of miRNA-34 and miRNA-203 by Snail as a part of the EMT program in cells [42,43] that leads to cancer progression. Thus, our analysis of the differences in individual miRNA expression shows that EVs released from cells in early EMT stage can carry miRNAs that promote cell migration and invasion of CRC cells and are associated with poor prognosis for colorectal cancer. Further, the positive regulation of STAT3 is the most significant GO term for Biological Process (Figure S3). Cytokine-driven JAK/STAT3 pathway plays an important role in the processes of signal transduction and is associated with the hyperproliferative and invasive phenotype of CRC cells [44].

Tumors induced by injection of HT29-Snail, as compared to control HT20-pcDNA cells, tend to have a lower rate of growth (Figure 3A). This observation is in an agreement with the inhibition of cell proliferation within the growing tumor due to the cell EMT progression caused by Snail [18]. At the same time we have observed the increased amount of MCP-1/CCL2 in the plasma of mice bearing the tumours induced by HT29-Snail. These data are in agreement with the earlier findings suggesting that Snail, as EMT inducer, can induce MCP-1/CCL2 production [45]. MCP-1/CCL2 is a potent chemoattractant for circulating blood monocytes via binding to its receptor CCR2 but is not an effective chemoattractant for differentiated monocyte-derived macrophages [46,47]. In parallel with the results of inhibited tumour growth and increased production of MCP-1/CCL2 we have observed the increased number of early interstitial pneumonias in mice injected with HT29-Snail cells, as compared to HT29-pcDNA cells (Table 1, group IV and II). Additionally, almost all mice bearing tumour induced by HT29-Snail cells that were also injected with EVs released from HT29-Snail cells, presented the appearance of early interstitial pneumonias (Table 1, group VI), while injection of HT29-pcDNA-EV had no effect (Table 1, group V and IV, respectively). As HT29-Snail-EVs were injected only into the group that was bearing tumours induced by injection of HT29-Snail cells, we cannot exclude the possibility that HT29-Snail-EVs would have an effect on control (HT29-pcDNA) mice and that remains to be elucidated.

Our in vitro findings (Figure 5) suggest that EVs released from HT29-Snail cells that are in an early EMT stage affect macrophages differently than the EVs released from epithelial-like HT29 cells. We observed the inhibition of random motility of macrophages that were treated with HT29-Snail-EVs cells as compared to control EVs released from HT29-pcDNA cells. Thus, the macrophages that are affected by HT29-Snail-EVs may become retained in pre-metastatic niche. We have also found the differences in the secretion of IL-8 by macrophages (Figure 5B,C) that may be attributed to the variability in EVs miRNA profiles. We found for example that miR-142-5p is downregulated in EVs released from HT29-Snail 8 and -17, so relatively there is higher amount of miR-142-5p in control EVs. High concentrations of miR-142-5p were observed in ulcerative colitis (UC), the inflammatory disease that frequently leads to development of colorectal cancer. MiR-142-5p levels were negatively correlated with the expression of suppressor of cytokine signalling 1 (SOCS1) in UC patients [48]. Further, miR-142-5p increased the secretion of IL-6 and IL-8 in TNFα-treated-HT29 [48]. Thus, we postulate that downregulation of IL-8 secretion macrophages treated with HT29-Snail-EVs might be associated to some extend with lower levels of miR-142-5p.

## 4. Materials and Methods

### 4.1. Cell Culture and Differentiation

The HT29 cell line (cells: colon, disease: colorectal adenocarcinoma) was obtained from American Type Culture Collection (Manassas, VA, USA) and cultured in McCoy’s 5A medium (LifeTechnologies, Waltham, MA, USA), supplemented with 10% FBS (LifeTechnologies) and antibiotics—streptomycin and penicillin (P/S) (Sigma-Aldrich, St. Louis, MO, USA), primocin (Invivogen, San Diego, CA, USA). The cells were periodically tested for mycoplasma using the PlasmoTest (Invivogen). For isolation of EVs released by HT29, serum-free medium with P/S was used to rule out the effect of exosomes of foetal bovine serum.

THP-1 monocyte/macrophage-like cell line from American Type Culture Collection (Manassas, VA, USA) was cultured in RPMI-1640 culture medium supplemented with 1 mM sodium pyruvate, 10% FBS, 0.05 mM 2-ME, P/S and primocin. For differentiation of THP-1 monocytes into THP-1-derived macrophages (TDM), cells were cultured with 20 ng/mL of phorbol ester (PMA) in culture medium for 48 h. Isolations of monocytes from healthy donors were performed as described previously [49]. To generate monocyte-derived macrophages (MDM) human monocytes were cultured 7 days with 10 ng/mL of GM-CSF (Thermo Scientific, Waltham, MA, USA) in RPMI-1640 supplemented with 10% human serum type AB from Sigma-Aldrich (St. Louis, MO, USA). All cell cultures were performed in a 90–95% humidified atmosphere of 5% CO_2_.

### 4.2. HT29 Stable Clone Generation and Isolation of EVs from Culture Supernatants

The pcDNA3.1 vector (Invitrogen, Carlsbad, CA, USA) and pcDNA3.1 vector expressing Snail was obtained from Prof. Muh-Hwa Yang (Institute of Clinical Medicine, National Yang-Ming University, Taipei, Taiwan). HT29 nucleofection and clone generation was performed as previously described [18]. Western blot showing the levels of Snail in clones -3, -8, and -17 is shown in Supplemental Figure S5. The HT29 cell line and HT29-pcDNA control clone were authenticated by ATCC using Short Tandem Repeat (STR) analysis. Extracellular vesicles were isolated by differential centrifugations and subsequent ultra-centrifugations as described previously [19]. HT29-Snail 3, 8, 17 and HT29-pcDNA clones were grown to 70–80% confluence on 15-cm dishes, washed three times with empty medium to remove vesicles present in FBS of culture medium and then cultured for 24 h to obtain conditioned medium. Next the medium was collected and centrifuged (350× *g* for 10 min) to remove floating cells. The supernatants were then collected and centrifuged at 2000× *g* for 20 min to remove APOs. Finally, EVs pellets were obtained after ultra-centrifugation for 1.5 h at 100,000× *g* using OPTIMA L-80 Ultracentrifuge and Type 45 Ti Rotor, Fixed Angle (Beckman Coulter, Inc., Brea, CA, USA). The pellets were next washed by diluting in PBS and centrifuging for 1.5 h at 100,000× *g*. All centrifugations were performed at 4 °C. Finally, EVs pellets were resuspend in PBS for Western blots or in appropriate media for functional experiments.

### 4.3. Nanoparticle Tracking Analysis (NTA)

EV size distribution and quantification of vesicles were analyzed by NTA using a NanoSight NS300 System (Malvern Panalytical Ltd., Malvern, UK) by a courtesy of the representative of company (A.P. Instruments, Warsaw, Poland).

### 4.4. Transmission Electron Microscopy (TEM)

TEM assay was used to evaluate the shape and size of EVs. Ten microliters of the sample were placed on 200-mesh copper grids with a carbon surface. The samples were negatively stained with 2% uranyl acetate for 1 min. and dried at room temperature. The transmission electron microscopy images were obtained using JEOL-1010 (Akishima, Japan).

### 4.5. miRNA Isolation from Extracellular Vesicles and miRNA Content Analysis

EVs pellets were treated with RNAze A (20 μg/mL in PBS). Total RNA was isolated and quality control of RNA was performed as described earlier [19]. Next-generation sequencing (NGS) analysis of the miRNAs was performed by Exiqon (https://www.exiqon.com/small-rna-ngs). For the comparisons between control EVs released from HT29-pcDNA and EVs released from clones HT29-Snail 3, -8, -17, the Benjamini-Hochberg FDR corrected *p*-values were calculated.

### 4.6. Animal Studies

The animal experiments were performed in the Center for Experimental Medicine Medical University of Bialystok (PL), in compliance with the Local Ethical Committee for Experiments on Animals in Olsztyn. Female CByJ.Cg- Foxn1<nu>/ccmdb mice (~20 g, 6–8 weeks old) were obtained from the same Center. Animals in group I were left as controls. The primary tumours were established by inoculating HT29-pcDNA or HT29-Snail 17 cells (1.5 × 10^6^/100 µL PBS) subcutaneously into the flank of the mice (group II and IV respectively). Tumour volumes were calculated: (length x width^2^)/2. Animals from group II and IV were additionally injected intravenously (tail vein) with 10 μg/mouse of indicated EVs (groups III, V and VI). Blood was collected from the retroorbital sinus (~50μL at day 7) and during the section of animals at the end of the procedure (day 28). Collected organs (liver, lungs, large intestine) were divided and fixed and stained with haematoxylin and eosin (H & E). Tumour growth was estimated by fitting each animal’s tumour growth to an exponential model. To fit data to this model, at first low volumes were truncated (up to 12 day) and log_10_ tumour volume versus time for each animal was plotted. The slopes and R^2^ values for the fits were calculated using linear regression [50].

### 4.7. PKH67 Labelling of Extracellular Vesicles and Their Uptake into TDM Cells

Extracellular vesicles were labelled using PKH67 Fluorescent Cell Linker kit (Sigma-Aldrich) according to the manufacturer’s instructions, with minor modifications [51]. After 4 h of PKH67-labelled EV incorporation into TDM, cells were fixed, treated with 0.1% Triton X-100 and incubated in sequence with Texas Red^®^-X phalloidin (F-actin marker) and Hoechst 33,342 (cell-permeant nuclear dye). The uptake was visualized using a confocal microscope (Nikon D-Eclipse C1) and analysed with EZ-C1 software.

### 4.8. Cell Motility Measurements

Measurements of cell motility were performed between the 7th and 25th hour after beginning of incubation of TDM with EVs using HoloMonitor M4 (Phase Holographic Imaging PHI AB, Lund, Sweden—Courtesy of the representative of company). Cell speed was calculated using the App Suite software of the same company. A plot of cell speed versus time was generated and the area under curve (AUC) was computed using GraphPad Prism 7.05 software.

### 4.9. Measurements of Cytokine Release

Macrophages were incubated with EVs in FBS-free RPMI-1640 culture medium for 24 h, the medium collected and centrifuged for 20 min at 18,000× *g*. IL-8 levels in the cells supernatants were analysed by flow cytometry according to the manufacturer’s procedure using the BD Cytometric Bead Array (CBA) Human Inflammatory Cytokines Kit. The BD Cytometric Bead Array (CBA) Mouse Inflammation Kit was used to measure MCP-1/CCL2 in the plasma of mice. Data were analysed using FCAP Array^TM^ Software Version 3.0 (BD Life Sciences—Biosciences, San Jose, CA, USA).

### 4.10. Statistical Analyses

Data are presented as mean ± SEM. All experiments were performed at least in triplicate. The Shapiro-Wilk test was used to confirm the Gaussian distributions of raw data. Analysis of variance (ANOVA) was used for multiple comparisons. The Kruskal-Wallis analysis was performed to test the differences between groups of data with non-normal distributions. For analyses of two groups, the appropriate Student’s *t* test (or the Welch’s test for unequal variances) was performed to test the differences between groups for normally distributed data. p value less than 0.05 was considered statistically significant. All statistical analysis was performed using GraphPad Prism 7.05 software.

## 5. Conclusions

We conclude that Snail that modifies CRC cells towards a more invasive phenotype, can also alter microRNA cargo of cell-released EVs. Thus, the content of cell-released EVs may serve as a biomarker that defines the stage of CRC and either Snail, or the different microRNAs that is carried by EVs to the destination sites, which serves as a pre-metastatic niche, may be a target to prevent cancer progression. We also point to the macrophages that may reside in the tumour pre-metastatic niche, as one of the possible EVs recipient cells that is modified during growing CRC invasiveness.

## Figures and Tables

**Table 1:** Incidence of lung infection in mice with tumours inflicted by injection of control HT29-pcDNA or HT29-Snail cells followed by injections of various EVs.

Group	Cell Injection Day 0	EV Injection Day 8, 12, 15, 18	Lung Infection Day 28
I	None	None	0/5 (0%)
II	HT29-pcDNA	None	0/5 (0%)
III	HT29-pcDNA	HT29-pcDNA	1/5 (20%)
IV	HT29-Snail17	None	2/5 (40%)
V	HT29-Snail17	HT29-pcDNA	2/5 (40%)
VI	HT29-Snail17	HT29-Snail17	4/5 (80%)

## Data Availability

Publicly available datasets were analyzed in this study. This data can be found here: https://www.ncbi.nlm.nih.gov/sra/PRJNA674779.

## Supplementary Materials

The following are available online at https://www.mdpi.com/2072-6694/13/2/172/s1, Figure S1: Western blot of EVs markers, Figure S2: Global efficiency of miRNA processing, Figure S3: Gene Ontology Enrichment Analysis, Figure S4: Cell viability assay, Figure S5: Snail transcription factor expression in HT29 clones, Table S1: Clone-specific miRNA evaluated during analysis of differentially expressed extracellular vesicles miRNA (fold change ≤ −2.0 for down-regulation and ≥ 2.0 for up-regulation) between each clone overexpressing Snail and control clone, Table S2: Differentially expressed miRNA evaluated during analysis of extracellular vesicles miRNA between each clone overexpressing Snail and control clone, Table S3: The growth rate of tumours and MCP-1/CCL2 levels in plasma of mice injected s.c. with of control HT29-pcDNA or HT29-Snail cells followed by i.v. injections of various EVs.
