# Supplementary material for: Snail Overexpression Alters the microRNA Content of Extracellular Vesicles Released from HT29 Colorectal Cancer Cells and Activates Pro-Inflammatory State In Vivo
Source: Cancers (Basel). 2021 Jan 6;13(2):172. doi: 10.3390/cancers13020172 (PMC7830966; doi:10.3390/cancers13020172)

CD63 in HT29-pcDNA (non-reducing conditions, ~55kDa)

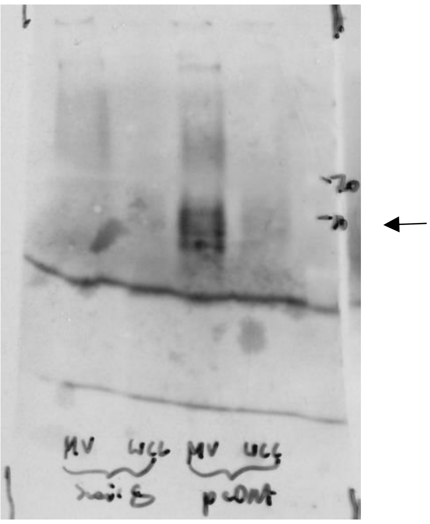

CD63 in HT29-Snail 17(non-reducing conditions, ~55kDa)

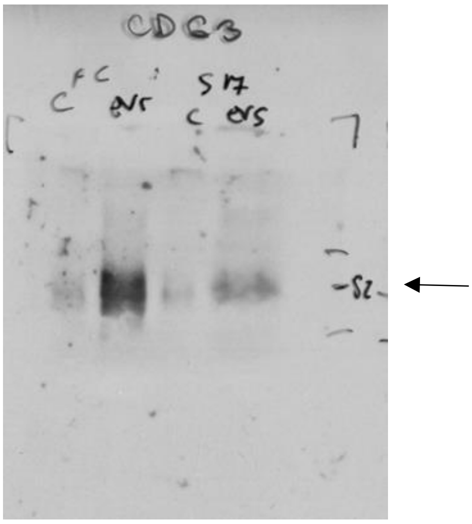

CD9 in HT29-pcDNA (25kDa)

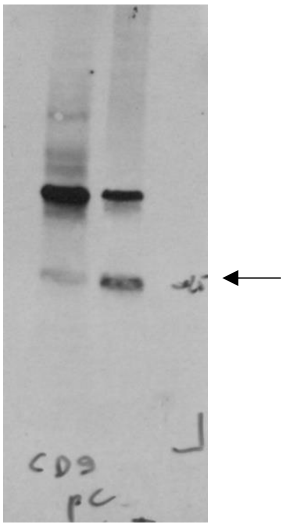

CD9 in HT29-Snail 17 (25kDa)

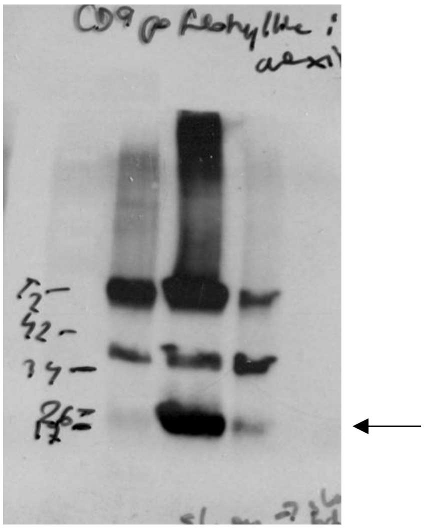

Flotillin-1 in HT29-pcDNA (49kDa)

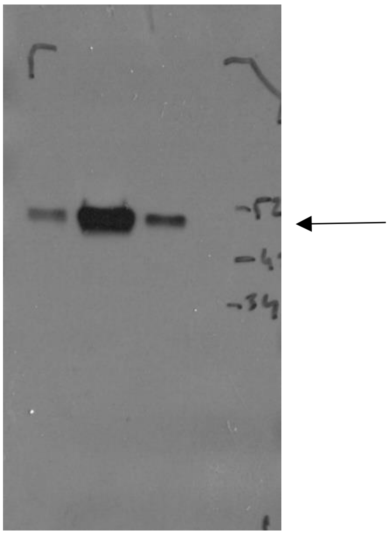

Flotillin-1 in HT29-Snail 17 (49kDa)

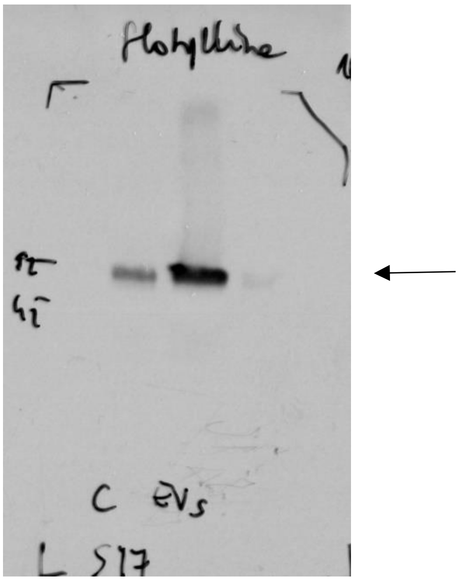

HSP70 in HT29-pcDNA and HT29-Snail 17

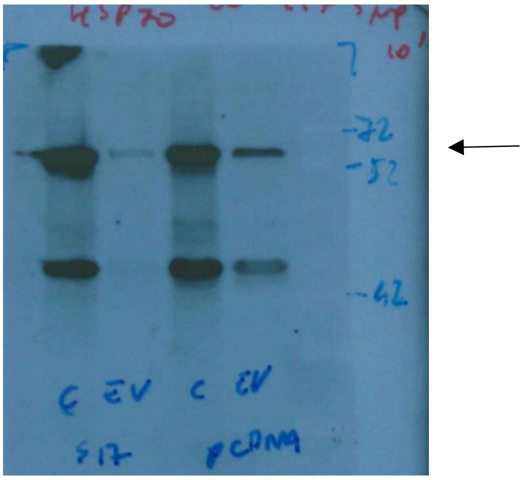

Annexin V in HT29-pcDNA (34kDa)

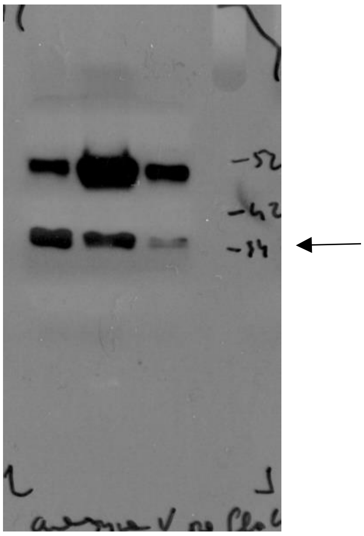

Annexin V in HT29-Snail 17 (34kDa)

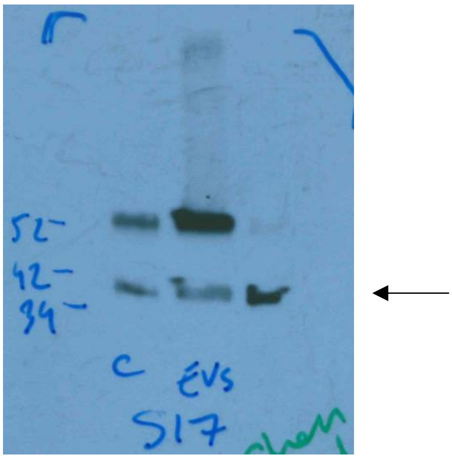

Cytochrome c in HT29-pcDNA and HT29-Snail 17 (14kDa)

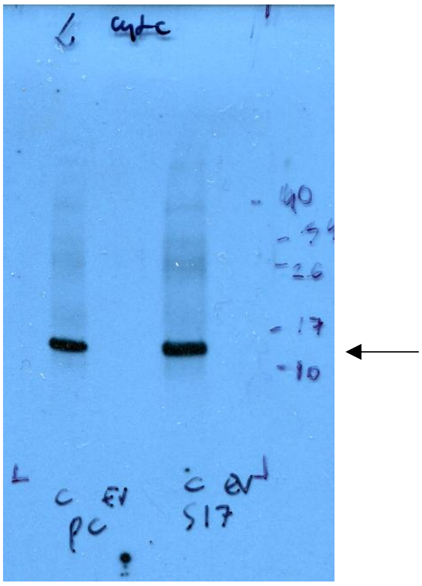

GM130 in HT29-pcDNA and HT29-Snail 17(130kDa)

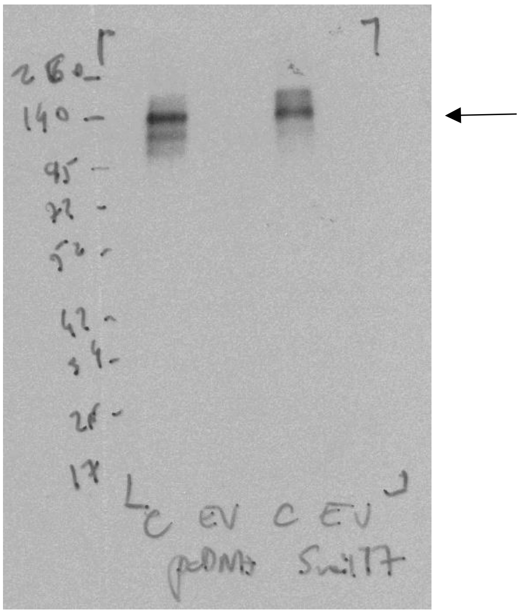

Snail in HT29-pcDNA and HT29-Snail clones (30 kDa)

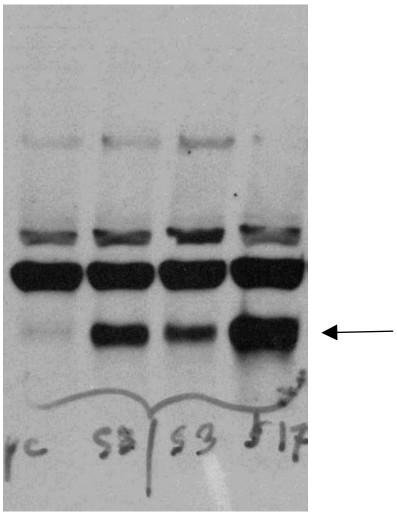

Supplement: Supplementary file 1 [file cancers-13-00172-s001.zip › cancers-977788 supplementary/cancers-977788 revised original WB.pdf]
