# Supplementary material for: Snail Overexpression Alters the microRNA Content of Extracellular Vesicles Released from HT29 Colorectal Cancer Cells and Activates Pro-Inflammatory State In Vivo
Source: Cancers (Basel). 2021 Jan 6;13(2):172. doi: 10.3390/cancers13020172 (PMC7830966; doi:10.3390/cancers13020172)
Supplement: Supplementary file 1 [file cancers-13-00172-s001.zip › cancers-977788 supplementary/cancers-977788 supplementary.pdf]

cancers

MDPI

Supplementary Materials: Snail overexpression alters the microRNA content of extracellular vesicles released from HT29 colorectal cancer cells and activates pro-inflammatory state in vivo

Izabela Papiewska-Pająk, Patrycja Przygodzka1 Damian Krzyżanowski, Kamila Soboska, Izabela Szulc-Kiełbik, Olga Stasikowska-Kanicka, Joanna Boncela, Małgorzata Wągrowska-Danilewicz, M. Anna Kowalska

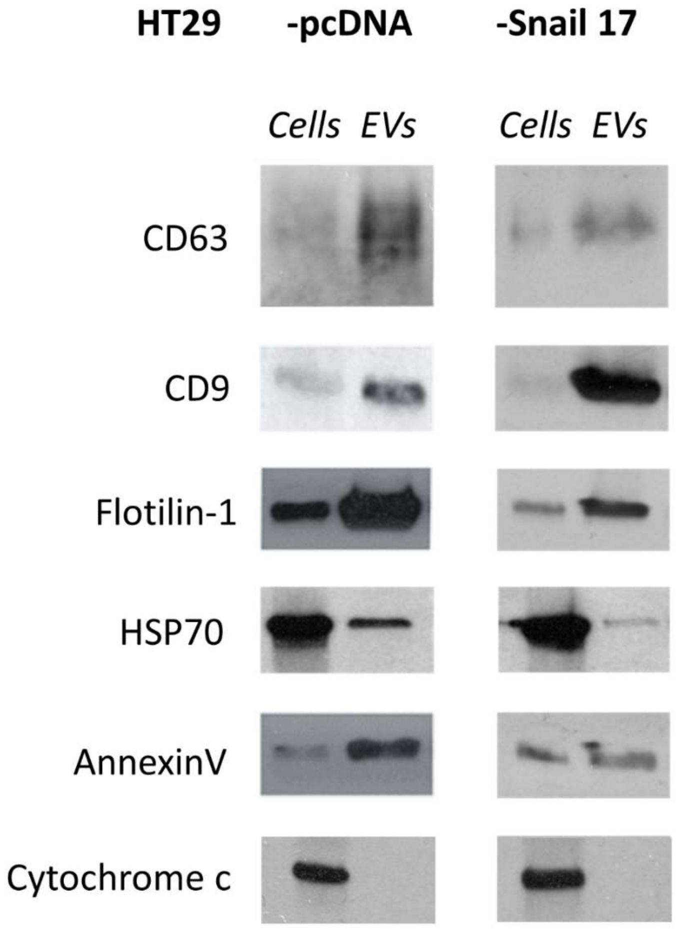

Figure S1. Western blot of EVs markers. EVs released from HT29-pcDNA and HT29-Snail 17 cells as compared to lysates of cells of origin. Proteins from cells and EVs were extracted with RIPA buffer or non-reducing buffer for CD63 with the Halt protease inhibitor cocktail (Thermo Scientific, Waltham, MA, USA) [30]. The primary (rabbit) and secondary (HRP-conjugated) antibodies for EVs markers were from Cell Signaling Technology Inc., and mouse anti-CD63 from Abcam (Cambridge, UK). Enhanced chemiluminescence kit was from Thermo Fisher Scientific and Kodak BioMax Light Film from Eastman Kodak.

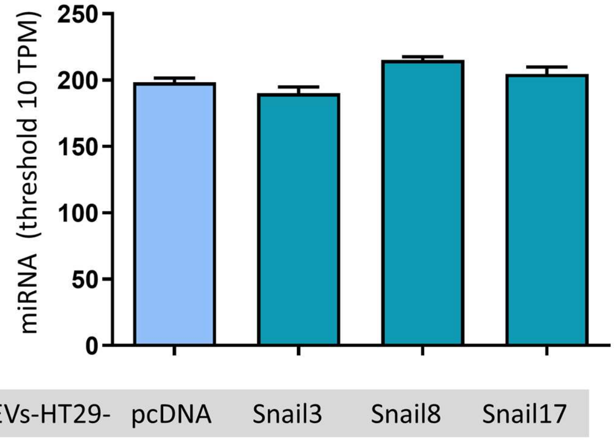

Figure S2. Global efficiency of miRNA processing. Number of identified known microRNAs with number of counts (>10TPM per sample) in HT29-pcDNA-EVs and HT29-Snail-EVs. Normalization procedure with TPM (Tags Per Million) was applied in Next Generation Sequencing (NGS) analysis. The number of reads that map to a particular RNA species is divided by the total number of mapped reads in the sample and subsequently multiplied by ten millions. That corrects for the sequencing depth and provides a very transparent measure of quantity for each RNA species. Snail upregulation in HT29 cells has no effect on the global efficiency of miRNA processing.

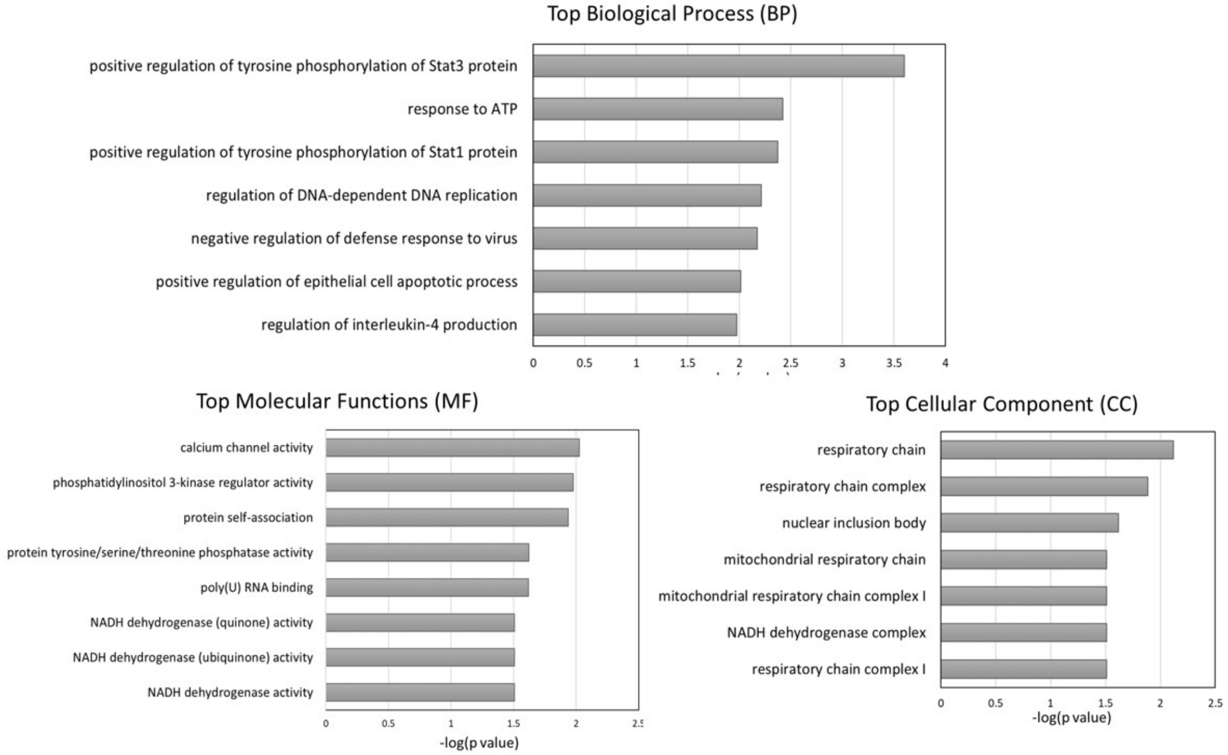

Figure S3. Gene Ontology Enrichment Analysis. The most significant GO terms for the microRNAs found to be differentially expressed between EVs released from HT29-Snail and HT29-pcDNA and their corresponding annotations for Biological Process, Molecular Function and Cellular Component. p-value < 0.05.

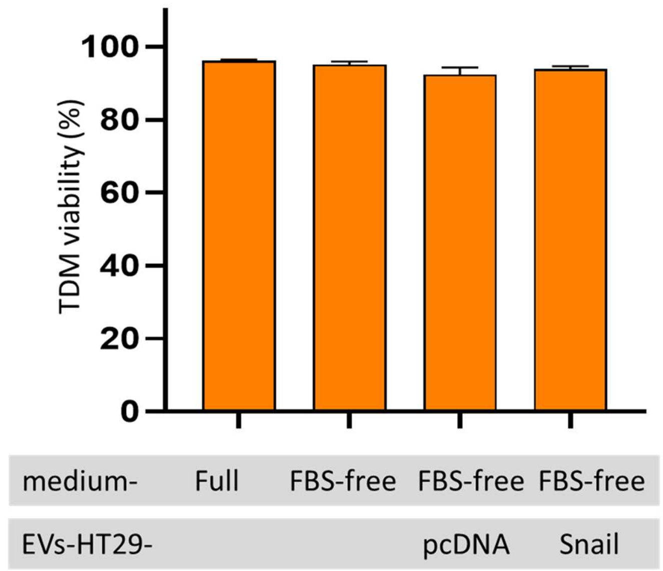

Figure S4. Cell viability assay. PI incorporation was analysed in THP-1-derived macrophages (TDM) incubated for 24 h with EVs according to the manufacturer's protocol using Becton Dickinson LSR II flow cytometer with BD FACS Diva software.

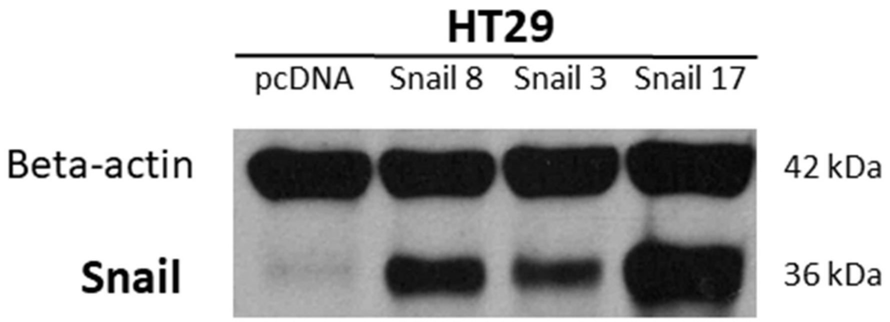

Figure S5. Snail transcription factor expression in the HT29 clones. After HT29 stable clone generation Snail expression was verified through Western blot analysis showing moderate (clone 3) and high (clone 8 and 17) Snail overexpression level. Proteins from cells were extracted with NP-40 lysis buffer with the Halt protease inhibitor cocktail (Thermo Scientific, Waltham,MA, USA). The equal amount of protein extracts (concentration measured BCA method (Pierce/Thermo Scientific, Waltham, MA, USA)) was subjected to SDS-PAGE analysis, transferred onto PVDF membranes (BioRad, Hercules, CA, USA) and blotted with mouse anti-Snail antibody (Cell Signaling Tech, Danvers,MA,USA). The control antibody, rabbit anti-β-actin was obtained from Abcam (Cambridge, UK). Secondary HRP-conjugated antibody was from Santa Cruz Biotechnology, Dallas, TX, USA, enhanced chemiluminescence kit from Thermo Scientific,Waltham,MA, USA, and Kodak BioMax Light Film from Eastman Kodak, Rochester, NY, USA.

Table S1. Clone-specific miRNA evaluated during analysis of differentially expressed extracellular vesicles miRNA (fold change ≤ −2.0 for down-regulation and ≥ 2.0 for up-regulation) between each clone overexpressing Snail and control clone. miRs marked in red are discussed in the text.

EVs-Snail 3-Specific miRNA	EVs-Snail 8-Specific miRNA	EVs-Snail 17-Specific miRNA
	Down-regulated, Fold change ≤ −2.0	
hsa-miR-146a-5p	hsa-miR-455-3p	hsa-miR-1266-5p
hsa-miR-340-5p	hsa-miR-455-5p	hsa-miR-150-5p
hsa-miR-651-5p		hsa-miR-203a
		hsa-miR-203b-3p
		hsa-miR-338-3p

		hsa-miR-378a-3p
		hsa-miR-378c
		hsa-miR-378d
		hsa-miR-378i
		hsa-miR-549a
Up-regulated, Fold change ≥ 2.0		
hsa-miR-1278	hsa-miR-1323	hsa-miR-126-3p
hsa-miR-27a-5p	hsa-miR-146a-5p	hsa-miR-149-5p
hsa-miR-301a-5p	hsa-miR-372-3p	hsa-miR-152-3p
hsa-miR-3065-5p	hsa-miR-512-3p	hsa-miR-181d-5p
hsa-miR-338-5p	hsa-miR-516b-5p	hsa-miR-193a-5p
hsa-miR-99b-3p	hsa-miR-584-5p	hsa-miR-193b-3p
hsa-miR-99b-5p	hsa-miR-6716-3p	hsa-miR-197-3p
	hsa-miR-874-3p	hsa-miR-221-5p
		hsa-miR-450b-5p
		hsa-miR-483-5p
		hsa-miR-582-5p
		hsa-miR-590-3p
		hsa-miR-934

Table S2. Differentially expressed miRNA evaluated during analysis of extracellular vesicles miRNA between each clone overexpressing Snail and control clone. miRs marked in red are discussed in the text.

**EVs-Snail 3-Specific miRNA**	**EVs-Snail 8-Specific miRNA**	**EVs-Snail 17-Specific miRNA**
Down-regulated, Fold change between −2.0 and −1.5		
hsa-miR-192-5p	hsa-miR-141-3p	hsa-miR-1303
hsa-miR-194-5p	hsa-miR-378c	hsa-miR-139-5p
hsa-miR-203b-3p	hsa-miR-378d	hsa-miR-194-3p
hsa-miR-215-5p		
hsa-miR-340-3p		
hsa-miR-148a-3p		
	hsa-miR-200a-3p	
hsa-miR-34a-5p		hsa-miR-34a-5p
Up-regulated, Fold change between 1.5 and 2.0		
hsa-let-7e-3p	hsa-miR-149-5p	hsa-miR-222-3p
hsa-miR-125a-3p	hsa-miR-193b-3p	hsa-miR-32-5p
hsa-miR-1304-3p	hsa-miR-335-3p	hsa-miR-584-5p
hsa-miR-330-3p	hsa-miR-934	hsa-miR-874-3p
hsa-miR-3613-5p	hsa-miR-99b-3p	
hsa-miR-3615	hsa-miR-99b-5p	
hsa-miR-548o-3p		
hsa-miR-615-3p		
hsa-miR-641		
hsa-miR-125a-5p<br>hsa-miR-221-5p		
hsa-miR-221-3p		

Table S3. The growth rate of tumours and MCP-1/CCL2 levels in plasma of mice injected s.c. with of control HT29-pcDNA or HT29-Snail cells followed by i.v. injections of various EVs.

Cell injection<br>Day 0	EV Injection<br>Day 8, 12, 15, 18	Growth of Tumour<br>Rate/Slope<br>Day 12–28	MCP-1 in Plasma<br>ng/mL<br>Day 28
HT29-pcDNA	None	0.055 + 0.003	28.4 + 4.6
HT29-pcDNA	HT29-pcDNA	0.048 + 0.007	21.3 + 2.2
HT29-Snail17	None	0.044 + 0.004	39.2 + 7.2

HT29-Snail17	HT29-pcDNA	0.034 + 0.008	43.8 + 4.8
HT29-Snail17	HT29-Snail17	0.040 + 0.003	30.3 + 5.1
